# An overview of antimicrobial resistance surveillance among healthcare-associated pathogens in South Africa

**DOI:** 10.4102/ajlm.v7i2.741

**Published:** 2018-12-06

**Authors:** Ashika Singh-Moodley, Husna Ismail, Olga Perovic

**Affiliations:** 1 Centre for Healthcare-associated infections, Antimicrobial Resistance and Mycoses, National Institute for Communicable Diseases, Johannesburg, South Africa

## Abstract

Healthcare-associated infections are a serious public health concern resulting in morbidity and mortality particularly in developing countries. The lack of information from Africa, the increasing rates of antimicrobial resistance and the emergence of new resistance mechanisms intensifies this concern warranting the need for vigorous standardised surveillance platforms that produce reliable and accurate data which can be used for addressing these concerns. The implementation of national treatment guidelines, policies, antimicrobial stewardship programmes and infection prevention and control practices within healthcare institutions require a platform from which it can draw information and direct its approach. In this review, the importance of standardised surveillance systems, the challenges faced in the application of a surveillance system and the condition (existence and nonexistence) of such systems in African countries is discussed. This review also reports on some South African data.

## Introduction

Infections caused by healthcare-associated pathogens are a public health concern that results in morbidity and mortality particularly in developing countries.^1^ Patients reporting to South African hospitals (both the public and private healthcare sectors) are at high risk of acquiring healthcare-associated infections and the costs of managing these infections cause an additional burden.^2^ The inappropriate use and overuse of antimicrobial agents leads to the selection of antimicrobial-resistant organisms and since these agents play a critical role in healthcare, increasing rates of resistance creates a serious threat in healthcare settings.^3^ The World Health Organization (WHO) released its first global antimicrobial resistance surveillance report in 2014 which stated that resistance to a wide range of antimicrobial agents is increasing in all six WHO regions. This report also highlighted that there was a significant gap in surveillance data and a lack of standards for methodology, data sharing and coordination in numerous countries worldwide, stressing the importance of and need for clearly defined, standardised surveillance systems.^4^ The Centre for Disease Control and Prevention (CDC) defines surveillance as the:

ongoing systematic collection, analysis and interpretation of health data essential to planning, implementation and evaluation of public health practice, closely integrated with the timely dissemination of these data to those who need to know.^5^

In a hospital environment, surveillance is an important tool that requires the implementation of an effective and integrated programme encompassing antimicrobial resistance, an antimicrobial stewardship programme and infection prevention and control.^2,6^ The extent of antimicrobial resistance in healthcare-associated pathogens needs to be established. However, it is challenging for healthcare facilities to perform surveillance due to the lack of standardised definitions for healthcare-associated infections, data analysts, information technology (IT) structure and staff trained on infection prevention, particularly in resource-limited settings like sub-Saharan Africa.^7,8^ A systematic review of 190 antimicrobial resistance surveillance studies conducted in sub-Saharan Africa from January 1990 to January 2013 by Leopold and colleagues in 2014, further highlighted the flaws in currently available data and the challenges experienced when implementing antimicrobial resistance surveillance.^9^ Although South Africa has greater resources as compared to the rest of Africa, the quality of these programmes varies across institutions and standardised surveillance systems are not in place in most South African healthcare facilities.^2^ Nevertheless, the South African Society for Clinical Microbiology (SASCM) has published yearly surveillance reports from both the public and private healthcare sectors.^10^ Although reports are not accessible in real time, the data are made available. The latest accessible data from the public healthcare sector reported on bloodstream infections over the period January to December 2015 for Gram-negative organisms (*Acinetobacter baumannii* complex, *Enterobacter cloacae* complex, *Escherichia coli, Klebsiella pneumoniae* and *Pseudomonas aeruginosa*) and Gram-positive organisms (*Staphylococcus aureus, Enterococcus faecalis* and *Enterococcus faecium*) from five provinces (Gauteng, KwaZulu-Natal, Free State, Western Cape and Eastern Cape). SASCM extracted routine data from sentinel sites predominantly from large academic hospitals from an electronic database.^11^

SASCM also reported information from the private healthcare sector for the period January to December 2013 from urine and bloodstream infections from the same five provinces.^12^

Antimicrobial susceptibility for each of the pathogens was reported from both the public and private healthcare sectors in the country during these periods. The South African National Department of Health attributed the weaknesses of surveillance and reporting activities to a number of factors. These included the low number of trained microbiologists outside of major urban centres, limited funding, the lack of a national electronic prescribing system and lack of linkage to pharmacy, clinical and laboratory data systems in institutions resulting in incomplete and variably reported information on antimicrobial resistance and consumption.^13^ In the latest activities from the South African Department of Health, a national resistance map was released, and combined data for both public and private healthcare sectors on antimicrobial susceptibility levels are available for the first time.^14^

An example of a successful surveillance programme is the European Antimicrobial Resistance Surveillance Scheme (EARSS), which was developed in 1999 and monitored two bacterial pathogen types (*Staphylococcus aureus* and *Streptococcus pneumoniae*) in 12 laboratories. By 2015, the number of bacterial pathogens monitored increased to eight and included over 900 laboratories.^15,16^ From this, it is clear that it is important to start off with a manageable number of sites and provide good quality data that can then be expounded.

In an effort to improve worldwide antimicrobial resistance surveillance, the Global Antimicrobial Resistance Surveillance System (GLASS) was developed to support the Global Action Plan (GAP) on antimicrobial resistance. It is coordinated with the national action plans of countries and aims at enabling countries to generate antimicrobial resistance data that is standardised, comparable and validated. GLASS combines patient, laboratory and epidemiological surveillance information which when together can aid in understanding the extent of antimicrobial resistance in individual and susceptible populations worldwide.^17^ Currently, the GLASS early implementation manual focuses on selected bacterial pathogens together with their selected antimicrobial combinations from four clinical specimens.^17^ While surveillance systems have been established in Europe, Latin America, Central Asia and Eastern Europe, they are lacking in developing countries. The United Kingdom Department of Health therefore launched the Fleming Fund, aligned with WHO and GLASS, to support these countries in developing antimicrobial resistance surveillance systems.^18^ The African continent and Asia are included in this initiative. In addition, the African Society for Laboratory Medicine (ASLM), together with other international partners, is in the process of developing a holistic stepwise framework to coordinate laboratory-based antimicrobial resistance surveillance in Africa, including South Africa.^19,20^

## Surveillance of antimicrobial resistance in healthcare-associated pathogens in Africa

According to the WHO Global Report on Surveillance,^4^ antimicrobial resistance is increasing in the African region, and there have been reports of a significant number of antimicrobial-resistant bacteria that are transmissible in the hospital and in the community. Since surveillance of antimicrobial-resistant bacteria is performed only in a few African countries, there is a paucity of accurate and reliable information and consequently limited data concerning the true extent of the problem. An additional challenge noted during an external quality assessment of public health laboratories in Africa^21^ showed that many countries experience problems with performing antimicrobial susceptibility testing. From this external quality assessment, a question on the consistency and accuracy of antimicrobial susceptibility testing data arises. Furthermore, although some countries have established surveillance programmes, there is a lack of a formal framework across the region.^4,21^ As part of the WHO’s GAP on antimicrobial resistance, the WHO has sent an open call for countries to enrol in the GLASS to participate in a structured surveillance programme and provide reliable and complete demographic data.^22^ For a national laboratory-based surveillance programme, government commitment and support is non-negotiable. This requires the drafting of policies and strategies and the securing of resources both, financial and human. GLASS participation also requires the establishment of a coordinating centre which will systematically collect, analyse and share data nationally and internationally. Participation also requires a regional reference laboratory which will provide technical support including training, capacity building and strategy advice.^20^ A review of 12 published articles from 2005 to May 2015 on antimicrobial resistance in East Africa^23^ showed that resistance to commonly used antimicrobial agents was prevalent and that multidrug resistance was increasing in the region. There was scarcity of data on the prevalence of antimicrobial resistance and hospital-acquired infections in developing countries in East Africa and the authors suggest that intensive investigation and surveillance is warranted.^23^ This was again indirectly reiterated in a systematic review published in 2017 of antibiotic-resistant *Escherichia coli* and *Salmonella* data from 2004 to early 2015 in Tanzanian healthcare settings.^24^From this review, a significant increase in resistance was observed. The authors began by stating in their abstract that reliable data are limited and recommend that proactive strategies in antimicrobial stewardship and infection control measures are crucial and must be implemented^24^, all of which can be solved by establishing a structured surveillance system.

## Surveillance of antimicrobial resistance in healthcare-associated pathogens in South Africa

In South Africa there is a high burden of infectious diseases consisting of a significant population that are of bacterial origin^25^. A prominent increase in extended-spectrum beta-lactamase (ESBL) production and emergence of carbapenemase production in *Klebsiella pneumoniae* and *Enterobacter* spp., multidrug resistance and an increase of carbapenemase production in *Acinetobacter baumannii* and *Pseudomonas aeruginosa*, an increase in multidrug-resistant *Escherichia coli* and resistance in Gram-positive isolates with a decline in methicillin-resistant *Staphylococcus aureus* (MRSA) and a steady increase in vancomycin-resistant enterococci (VRE) have been noted in various reports^26,27,28,29,30,31,32^ making use of various methodologies. The need for a systematic and consistent approach in selecting antimicrobials for the treatment of these infections is essential. An established standardised surveillance system will provide information on causative pathogens and antimicrobial susceptibility patterns of these pathogens. In addition, the antimicrobial spectrum of a selected agent, the use of appropriate dosing schedules based on the selected agent’s pharmacokinetic and pharmacodynamic properties and pharmacological considerations in the patient should be included. An intervention strategy such as directing therapy to narrow-spectrum agents once microbiology results become available should also be established as well as the routes of transmission when a resistant strain is recovered from more than one individual. Once this information is known, an intervention can be introduced and subsequently proved effective.^2,7^

A report by Crowther-Gibson and colleagues in 2011^25^ aimed at summarising surveillance efforts in South Africa. This report reviewed the national burden of disease and levels of antimicrobial resistance in common bacterial infections and showed that antimicrobial resistance in healthcare-associated infections varied among antimicrobial agents in the public and private healthcare sectors. This report indicated the need for continuous monitoring as an effort to observe and control the spread of resistance. Limitations noted were that the causes of illness and deaths were not well documented, a common occurrence in low-resource countries. Distinguishing viral from bacterial diseases requires a level of detail that did not exist in the majority of the cases.^25^

Other efforts to publish South African data is the SENTRY Antimicrobial Surveillance Programme which is an international programme documenting antimicrobial resistance in predominantly healthcare-associated pathogens.^33,34,35,36,37,38,39,40,41,42,43^ Surveillance data, however, do not report findings from South Africa exclusively hence findings specific to the country cannot be extracted.

Antimicrobial resistance surveillance in healthcare-associated infections is particularly important because it is crucial to understand the dynamics in a specific healthcare setting. However, as patients are often transferred between wards and hospitals it becomes problematic to establish a system that would work in all healthcare settings due to differences in staff complement, antimicrobial stewardship programmes and infection prevention and control practices. These differences highlight this challenge and is further reiterated in a 6-month surveillance study conducted in Tygerberg Children’s Hospital, Cape Town, in 2015 whose aim was to evaluate three surveillance methods. Point prevalence surveys, laboratory surveillance and tracking of antimicrobial prescriptions were evaluated against the reference method which was prospective clinical healthcare-associated infection surveillance. While a combination of antimicrobial prescription tracking and laboratory surveillance produced the best results in their setting, the authors concluded that healthcare facilities should select a suitable method subject to available resources and practice context.^44^ However, as indicated repeatedly in the current review, the problem that exists with such a recommendation is that methodologies are not standardised and in order to obtain a holistic picture of antimicrobial resistance in healthcare-associated infections in South Africa, standardised methods need to be implemented.

Our group at the Antimicrobial Resistance Laboratory, which is a national reference laboratory at the National Institute for Communicable Diseases (NICD), established and commenced (through the GERMS-SA platform) a standardised laboratory-based antimicrobial resistance surveillance (LARS) programme of pathogens causing healthcare-associated infections such as the ESKAPE pathogens (*Enterococcus faecium, Staphylococcus aureus, Klebsiella pnemoniae, Acinetobacter baumanii, Pseudomonas aeruginosa* and *Enterobacter* species) at sentinel sites in 2010 and electronic surveillance from routine laboratories in 2013. The primary objectives of the LARS programme were to determine the number of cases reported from selected hospitals for selected pathogens, to describe antimicrobial susceptibility of the significant treatment regimens for the pathogens^45^ and a secondary objective was molecular characterisation of resistance genes.^26,27,30^

In 2014, antimicrobial resistance surveillance data on *Klebsiella pneumoniae* isolates from patients with bacteraemia were published^30^; isolates were submitted by sentinel laboratories in five regions of South Africa from mid-2010 to mid-2012. This included 13 academic centres serving the public healthcare sector in the Gauteng, KwaZulu-Natal, Free State, Limpopo and Western Cape provinces. Findings from this study showed that of the 2774 isolates, 1895 (68%) expressed ESBLs and displayed resistance to cefotaxime, ceftazidime and cefepime. More so, 46% of all isolates were resistant to ciprofloxacin and 33% to piperacillin-tazobactam. Susceptibility to aminoglycosides was variable: an average of 95% were susceptible to amikacin but only 31% were susceptible to tobramycin and gentamicin. Susceptibility to ciprofloxacin (53%) was lower than to levofloxacin (75%) while susceptibility to cefoxitin was higher (87%). The minimal inhibitory concentration (MIC) measures, the lowest concentration required to inhibit the growth of 50% (MIC_50_) and 90% (MIC_90_) of the isolates, were stable over the 3-year period and there was a small but statistically significant trend (*p* < 0.001–0.41) towards a decrease in susceptibility to many antimicrobial agents. Molecular characterisation was performed on 270 phenotypically ESBL-producing isolates. The presence of ESBLs (*bla*_CTX-M_, *bla*_SHV_ and *bla*_TEM_ genes) were confirmed in all 270 isolates with 93% of the isolates tested expressing more than one resistance gene. The majority of the isolates (95%) were phenotypically susceptible to the carbapenems tested, and no isolate contained *bla*_KPC_ or *bla*_NDM-1_, which were the only carbapenemases screened for. The authors stated that the high proportion of ESBL-producing *K. pneumoniae* isolates and the prevalence of ESBL genes is of great concern.^30^ More recently data from a cross-sectional study on carbapenem-resistant enterobacteriaceae (CRE) isolates showed a 1.9% prevalence of carbapenemases in blood culture isolates.^26^

Another publication from LARS-generated surveillance data investigated *Staphylococcus aureus* bacteraemia in academic hospitals from Gauteng, South Africa.^46^ In this study, the epidemiology of *S. aureus* bacteraemia was described and factors associated with MRSA infection were determined. Cases of *S. aureus* bacteraemia over 1 year (September 2012 to September 2013) were reported from three sentinel sites and detailed clinical information was collected. Statistical analysis included multivariable logistic regression to determine factors associated with MRSA infection and mortality. This study showed that 442 cases of *S. aureus* bacteraemia were reported and antimicrobial susceptibility testing was performed on 54% of the isolates (*n* = 240). Methicillin-resistant *Staphylococcus aureus* infection was noted in 36% (*n* = 86) of cases. Independent predictors of MRSA included a longer hospital stay before positive specimen collection, hospitalisation in the last year, human immunodeficiency virus (HIV) infection and antimicrobial use in the previous 2 months. The only independent predictor of mortality among cases with *S. aureus* bacteraemia was being elderly. Antimicrobial susceptibility testing results showed that MRSA isolates were non-susceptible to more antimicrobial agents compared to methicillin-susceptible *S. aureus* (MSSA) isolates. All isolates were susceptible to daptomycin and linezolid. Four isolates were non-susceptible to vancomycin and teicoplanin (MIC 16 mg/ml) while 13 cases (6%) (five MRSA and eight MSSA isolates) showed a susceptible breakpoint value (MIC 2 mg/ml). For molecular characterisation, SCC*mec* typing was performed on 82 MRSA isolates. Nine isolates were non-typeable; the most common SCC*mec* type was type III (56%), followed by type IV (29%) typically associated with hospital- and community-acquired infections, respectively. Type II accounted for only four isolates and no type I isolates were found. Clinical information was available for 140 cases. According to the authors, majority of patients (86%, 121/140) received one or more antimicrobial agents, 95 cases received empirical treatment while 42 received directed treatment. Of those that received empirical treatment (*n* = 95), a greater proportion of MSSA cases (95%, 55/58) received appropriate empirical therapy compared to MRSA cases (57%, 21/37) and this was statistically significant (*p* < 0.001). Of those that received directed treatment (*n* = 42), all MSSA cases (25/25) received appropriate directed therapy while this was true for only 59% (10/17) of MRSA cases; again, this was statistically significant (*p* < 0.001). This study demonstrated that antimicrobial prescription practices should be monitored and that knowledge of local epidemiology as well as factors predictive of MRSA infection is important as it will assist in guiding the use of appropriate empirical treatment. The importance of antimicrobial resistance surveillance as a public health measure was also highlighted.^46^

A more recent LARS study published in 2015^27^ investigating the prevalence and trends of *S. aureus* bacteraemia in 2709 hospitalised patients from 13 academic centres in South Africa in 2010 to 2012 examined antimicrobial resistance and the molecular epidemiology for laboratory-based surveillance. Forty-six per cent of cases were MRSA and there was a significant decline of MRSA from 53% to 40% over the 3-year period. Geographical distribution showed that MRSA was significantly higher in Gauteng compared to the other provinces and children younger than 5 years of age were associated with higher MRSA rates compared to all other age groups. Methicillin-resistant *Staphylococcus aureus* isolates were resistant to more classes of antimicrobial agents compared to MSSA isolates. Isolates were fully susceptible to glycopeptides, daptomycin, linezolid and quinupristin-dalfopristin. While ciprofloxacin and trimethoprim-sufamethoxazole resistance declined significantly over the surveillance period, resistance to macrolides, aminoglycosides, tetracycline, rifampin and mupirocin remained comparable. The MIC_50_ and MIC_90_ for all agents remained stable from 2010 to 2012. The most prevalent SCC*mec* type distribution was consistent with the previous study,^41^ with type III predominating (41%) followed by type IV (31%). Further molecular studies found 47 different *spa*-types with the five most common *spa*-types accounting for 87% of the isolates and were t037, t1257, t045, t064 and t012. The most common multilocus sequence type (MLST) was ST612 clonal complex 8 (CC8) (*n* = 7), followed by ST5 (CC5) (*n* = 4), ST36 (CC30) (*n* = 4) and ST239 (CC8) (*n* = 3). Overall, this study revealed the presence of a variety of hospital-acquired MRSA clones in South Africa and a dominance of a few clones, a result that was similar to previous findings in South Africa,^47,48,49^ indicating a slow evolution of circulating clonal types. This study demonstrated the importance of monitoring trends in resistance and molecular typing to detect changing epidemiological trends in antimicrobial resistance patterns.^27^ In South Africa, the Antimicrobial Resistance Laboratory at the NICD has been assigned as the WHO collaborating centre for antimicrobial resistance surveillance and in April 2017, responded to the first data call from the GLASS.^22^ GERMS-SA data for *S. aureus* isolated from blood culture specimens submitted from 2015 to 2016 showed a decrease in cefoxitin resistance, from 31.4% (*n* = 225/882) to 24.9% (*n* = 182/867) (unpublished data).

Secondary electronic surveillance for a select panel of bacteria (ESKAPE) modelled on the GLASS pathogen-antimicrobial combination is utilised for the purpose of complementing the well-established GERMS-SA surveillance system. Antimicrobial resistance data are channelled and formatted as antimicrobial resistance maps for both public and private healthcare sector laboratory results, and are made available on the NICD website.^14^

These antimicrobial resistance maps are summarised and published as annual reports on the Federation of Infectious Diseases Societies of Southern Africa (FIDSSA) website^10^. In 2016, *Klebsiella pneumoniae* isolates demonstrated 65% susceptibility to cefepime, 44% to piperacillin-tazobactam and 41% to gentamicin, while less than 30% of *Escherichia coli* isolates were non-susceptible to cephalosporins. For the non-fermentative Gram-negative bacteria, more than 80% of *Acinetobacter baumannii* isolates were non-susceptible to carbapenems, while 80% and 75% of *Pseudomonas aeruginosa* isolates were susceptible to cephalosporins and carbepenems respectively. For the Gram-positive bacteria, more than 99% of *Enterococcus faecalis* and *Enterococcus faecium* isolates demonstrated susceptibility to oxazolidinones and 1% and 5% of *E. faecalis* and *E. faecium* isolates demonstrated susceptibility to glycopeptides respectively. *Staphylococcus aureus* isolates demonstrated higher susceptibility to cloxacillin, from 65% in 2015 to 69% in 2016 (unpublished data). The findings from these studies and other ongoing studies which have not as yet been published show that South African data is being generated from the expanding surveillance programme in the hope of elucidating the situation of antimicrobial resistance in healthcare-associated pathogens in South Africa.

### Conclusion

Antimicrobial resistance surveillance in healthcare-associated settings, e.g. hospitals, long-term care facilities, dialysis clinics, etc., is essential in order to gain an understanding of the prevalent healthcare-associated pathogens such as the ESKAPE organisms, the antimicrobial resistance patterns of critical organisms and their mechanisms of resistance. National laboratory systems should support the surveillance for antimicrobial resistance and implement standardised practices, manuals and quality systems. In addition, this information will assist in the development of national treatment guidelines, inform policies and strategies for antimicrobial stewardship programmes and infection prevention and control. Due to the lack of reliable and accurate information in African countries, there is an urgent need for the implementation of standardised surveillance programmes.
